# Correction: A non-sacrificial method for the quantification of poly(ethylene glycol) grafting density on gold nanoparticles for applications in nanomedicine

**DOI:** 10.1039/d3sc90220j

**Published:** 2023-11-21

**Authors:** Jun Lu, Yao Xue, Rui Shi, Jing Kang, Chao-Yang Zhao, Ning-Ning Zhang, Chun-Yu Wang, Zhong-Yuan Lu, Kun Liu

**Affiliations:** a State Key Laboratory of Supramolecular Structure and Materials, College of Chemistry, Jilin University Changchun 130012 P. R. China kliu@jlu.edu.cn; b Institute of Theoretical Chemistry, Jilin University Changchun 130023 P. R. China luzhy@jlu.edu.cn

## Abstract

Correction for ‘A non-sacrificial method for the quantification of poly(ethylene glycol) grafting density on gold nanoparticles for applications in nanomedicine’ by Jun Lu *et al.*, *Chem. Sci.*, 2019, **10**, 2067–2074, https://doi.org/10.1039/C8SC02847H.

The authors regret that on page 9 of the ESI, the Stokes–Einstein equation and calculated diffusion coefficient (*D*) were incorrect. The corrected equation and calculated diffusion coefficient are shown here:
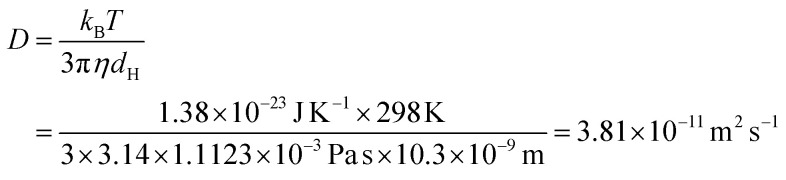


The value for the diffusion coefficient of pure gold nanoparticles (GNPs) on page 2068 of the main article should therefore be 3.81 × 10^−11^ m^2^ s^−1^.

The ESI available online has now been updated to reflect these changes.

The Royal Society of Chemistry apologises for these errors and any consequent inconvenience to authors and readers.

## Supplementary Material

